# Development of a dynamic myocardial perfusion phantom model for tracer kinetic measurements

**DOI:** 10.1186/s40658-022-00458-y

**Published:** 2022-04-25

**Authors:** Marije E. Kamphuis, Henny Kuipers, Jacqueline Verschoor, Johannes C. G. van Hespen, Marcel J. W. Greuter, Riemer H. J. A. Slart, Cornelis H. Slump

**Affiliations:** 1 Multi-Modality Medical Imaging (M3i) Group, Faculty of Science and Technology, Technical Medical Centre, University of Twente, P.O. Box 217, 7500 AE Enschede, The Netherlands; 2 Robotics and Mechatronics (RaM) Group, Faculty of Electrical Engineering Mathematics and Computer Science, University of Twente, Enschede, The Netherlands; 3 Department of Nuclear Medicine, Ziekenhuis Groep Twente, Hengelo, The Netherlands; 4 Medical Imaging Centre, Department of Radiology, University Medical Center Groningen, University of Groningen, Groningen, The Netherlands; 5 Medical Imaging Centre, Department of Nuclear Medicine and Molecular Imaging, University Medical Center Groningen, University of Groningen, Groningen, The Netherlands; 6 Biomedical Photonic Imaging Group, Faculty of Science and Technology, University of Twente, Enschede, The Netherlands

**Keywords:** Phantom model, Quantitative imaging, Perfusion, Myocardium, SPECT, Ground truth, Tracer kinetics

## Abstract

**Background:**

Absolute myocardial perfusion imaging (MPI) is beneficial in the diagnosis and prognosis of patients with suspected or known coronary artery disease. However, validation and standardization of perfusion estimates across centers is needed to ensure safe and adequate integration into the clinical workflow. Physical myocardial perfusion models can contribute to this clinical need as these can provide ground-truth validation of perfusion estimates in a simplified, though controlled setup. This work presents the design and realization of such a myocardial perfusion phantom and highlights initial performance testing of the overall phantom setup using dynamic single photon emission computed tomography.

**Results:**

Due to anatomical and (patho-)physiological representation in the 3D printed myocardial perfusion phantom, we were able to acquire 22 dynamic MPI datasets in which ^99m^Tc-labelled tracer kinetics was measured and analyzed using clinical MPI software. After phantom setup optimization, time activity curve analysis was executed for measurements with normal myocardial perfusion settings (1.5 mL/g/min) and with settings containing a regional or global perfusion deficit (0.8 mL/g/min). In these measurements, a specific amount of activated carbon was used to adsorb radiotracer in the simulated myocardial tissue. Such mimicking of myocardial tracer uptake and retention over time satisfactorily matched patient tracer kinetics. For normal perfusion levels, the absolute mean error between computed myocardial blood flow and ground-truth flow settings ranged between 0.1 and 0.4 mL/g/min.

**Conclusion:**

The presented myocardial perfusion phantom is a first step toward ground-truth validation of multimodal, absolute MPI applications in the clinical setting. Its dedicated and 3D printed design enables tracer kinetic measurement, including time activity curve and potentially compartmental myocardial blood flow analysis.

**Supplementary Information:**

The online version contains supplementary material available at 10.1186/s40658-022-00458-y.

## Background

Absolute, multimodal myocardial perfusion imaging (MPI) has become a clinically relevant research topic within the cardiac imaging community [1, 2]. Especially for positron emission tomography (PET), the advantages of quantitative assessment in addition to qualitative, visual evaluation have been demonstrated [3]. Quantification of myocardial blood flow (MBF) and myocardial flow reserve provides a substantial advantage for diagnostic and prognostic evaluation of suspected or established coronary artery disease [1–4]. Currently, these quantitative approaches are on the verge of being translated into the clinical workflow. In addition, similar approaches are also being explored for other imaging modalities, including computed tomography, magnetic resonance imaging and single photon emission computed tomography (SPECT). However, further efforts are necessary to standardize measures across clinical centers, radiotracers, equipment and software [4].

Our ultimate aim is to realize reference standards for validation and harmonization of absolute, multimodal MPI applications using physical perfusion models, i.e., hardware perfusion phantoms. Even though such models will never fully mimic actual patient anatomy and (patho-)physiology, it allows study of dynamic MPI applications in a simplified, although controlled environment [5]. Several left ventricular phantoms have been described in literature, in which various physiological states of perfusion can be simulated under static conditions without flow [6, 7]. In this, we observe an increasing trend in the use of 3D printing technology to obtain comprehensive representations of the heart [8–10]. Also, numerous perfusion phantoms have been proposed to facilitate physical flow standards for multimodal tissue perfusion imaging [11–14]. Nevertheless, current flow phantoms generally require in-house developed software for perfusion analysis. Evaluation of the entire absolute MPI application involves an adequate phantom to mimic flow dynamics on the one hand and to make clinical software believe the phantom resembles a human heart on the other (i.e., including anatomical representation).

This work describes an empirical approach toward the development of the Twente Myocardial perfusion (TMP) phantom, and initial performance evaluation in dynamic SPECT-MPI. In this paper, we introduce a unique way of mimicking radiotracer uptake and retention in the myocardial tissue. The main design goals are:

1. to make the phantom compatible with clinical perfusion analysis software, and
2. to incorporate mimicking of tracer kinetics (i.e., up to the level of ground-truth comparison with software-derived MBF estimation).

## Materials

The phantom setup has emerged from the redesign of a previously described myocardial perfusion phantom [15]. Phantom redesign concerned a novel way of tissue simulation, integration of coronary branches in the phantom model and improved sealing to prevent leakage. In addition, an important adjustment is the transition from an open to a closed flow circuit.

### Phantom design and realization

**Fig. 1:**
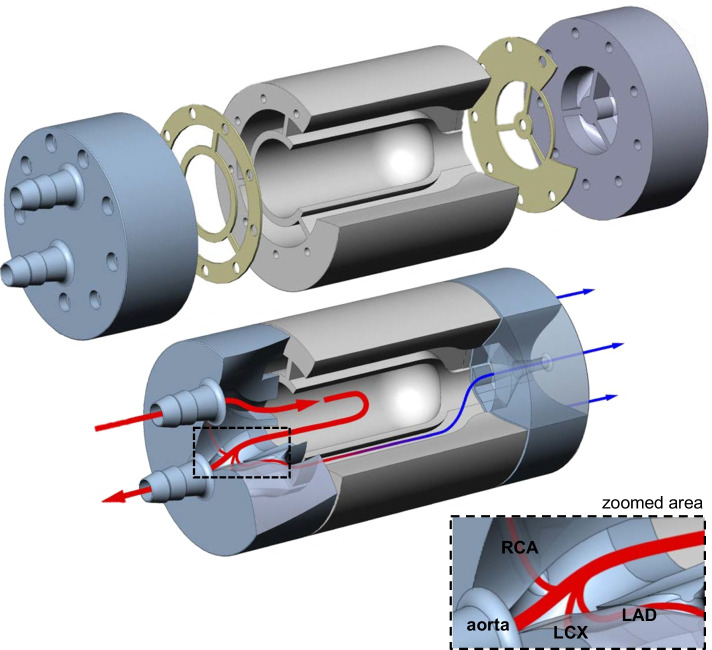
Exploded view (top) of phantom design and tailored cross section (bottom) to illustrate inner phantom connections. The arrows indicate flow direction and magnitude, and its color differentiates between arterial input (red) and venous output (blue). The middle phantom part comprises of the simulated left ventricular cavity and three surrounding myocardial segments. The two outer parts contain the in- and outlet connections to the flow circuit and the internal branches to the segments. These branches correspond to the main coronary arteries, i.e., the left anterior descending coronary artery (LAD), right coronary artery (RCA) and left circumflex coronary artery (LCX). The phantom parts are fastened together with nylon screws whereby silicone seals are placed in between the parts for waterproofing

**Fig. 2:**
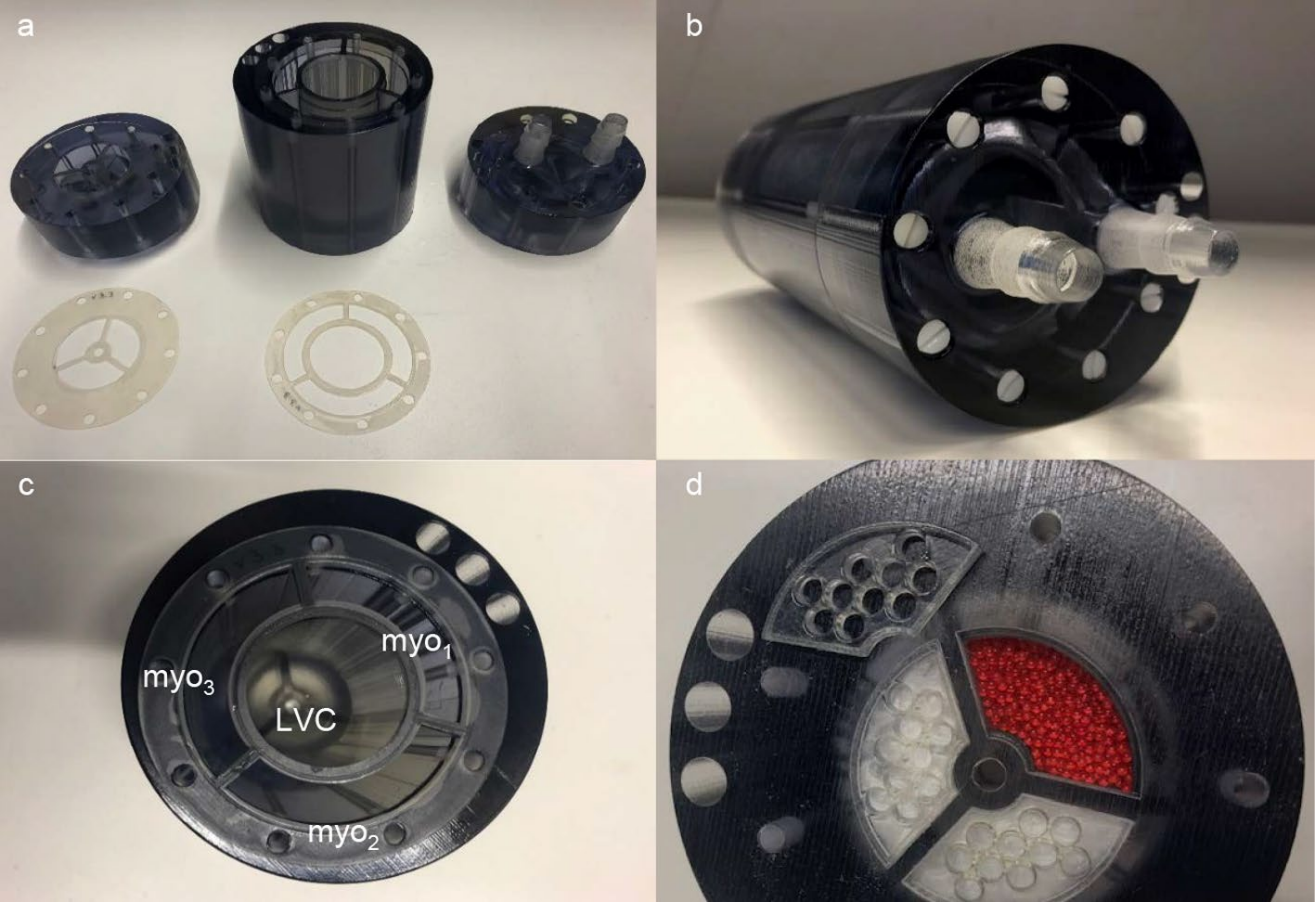
Overview of the realized myocardial perfusion phantom. **a** The individual 3D printed parts and silicone seals. In **b**, all pieces were assembled and secured by nylon screws (2 × 9). **c** The left ventricular cavity (LVC) and the three myocardial segments (myo_1–3_) are indicated in cross section. **d** Demonstrates fabrication of the myocardial inlays. The tissue mimicking material was enclosed by a glass wool layer and a 3D printed sieve before assembling all pieces

Figures 1 and 2 illustrate the designed and realized stationary myocardial perfusion phantom. All components were constructed with and designed for 3D printing (Objet260 Connex3, Stratasys Inc., Rehovot, Israel), enabling rapid prototyping. As visualized in the exploded view of Fig. 1, the phantom assembly consists of three cylindrical parts made of rigid transparent photopolymer material (VeroClear, Stratasys Inc., Rehovot, Israel). These parts were secured by nylon screws with silicone seals in between for waterproofing. The entire phantom cylinder fits as insert in an anthropomorphic thorax phantom (QRM, PTW company, Freiburg, Germany) for realistic x-ray attenuation during imaging [16].

The inner part includes the simulated left ventricular cavity (LVC) and surrounding myocardial tissue, while the two outer parts contain the branches to the myocardial tissue and the in- and outlet connections to the flow circuit. The LVC has an inner diameter of 38 mm and a length of 85 mm. The myocardium is mimicked by three identical segments, as depicted in Fig. 2c, d by the three circumferential cutouts (1 cm thick) from base to apex. These segments correspond to the three main coronary territories, i.e., regions supplied by the left anterior descending coronary artery (LAD), right coronary artery (RCA) and the left circumflex coronary artery (LCX). Each myocardial segment has an in- and outlet at the base and apex, respectively.

### Tissue mimicking

Distribution and retention of the injected radiotracer within the myocardial tissue is mimicked using sorption technology. Sorption occurs when a substance in a fluid is selectively transferred to insoluble, rigid particles [17]. In this way, the use of an adequate sorbent or mixture of sorbents can in essence mimic all kinds of desired radiotracer/contrast agent distribution and (temporary) trapping in a tissue perfusion phantom (our hypothesis). Commonly known adsorbents, i.e., activated carbon and zeolite, were in different quantities individually or as mixture (~ 50/50) blended with plastic beads before pouring into the empty myocardial segments. The plastic beads serve as tissue filling material. Figure 2d demonstrates the fabrication process of the myocardial tissue inlays. The tissue mimicking material was enclosed by a glass wool layer and 3D printed sieves to prevent the material from being carried into the fluid circuit. As part of this study, the composition of the myocardial segments was further explored empirically aiming to achieve adequate myocardial uptake of ^99m^Tc labeled pharmaceuticals for dynamic MPI-SPECT (see “Phantom measurements” section).

### Phantom flow setup

For several reasons, the phantom flow setup consists of a closed loop flow circuit. In this way, water can be circulated with the same flow over time and the circuit will not drain when turning the pump off in between measurements.

Tap water can be pumped at a continuous flow from the reservoir toward the TMP phantom using an external pump (Low voltage impeller pump, Barwig 02, Germany). Next, a radiotracer bolus can be administered via a clinical contrast injector (by Luer Lock connection). After injection, the radiotracer bolus is diluted and flows through the LVC where it is fully mixed with the water present in the LVC. The LVC outlet connects to the reservoir again but branches first to three myocardial segments. The segment outlets connect separately to the reservoir as well. The inserted flow sensors measure volume flux through the LVC (UF08B, ultrasonic flowmeter, Cynergy 3, UK) and myocardial segments (FCH-m-POM-LC, low flow turbine flowmeter, B.I.O-TECH, Germany). Four adjustable resistances can set the flow ratio between the returning parallel circuits. Hence, it becomes feasible to create a perfusion deficit in one or multiple myocardial segments within the TMP phantom by (partially) closing one or multiple tap(s). A pressure sensor (40PC series, Honeywell Inc., Freeport, Illinois) verifies whether the pump is still operating in the desired pressure range (kept below 1.0 bar). Pump steering and real-time sensor readout (sample frequency of 1 Hz) is achieved using in-house built hard- and software [18]. The latter is used to set the resistances accordingly before starting a measurement.

Finally, custom-built filters are positioned in all returning tubing to extract radioactive material from the water before re-entering the reservoir. In this way, controlled first pass radiotracer kinetics is envisaged without the necessity of a rather unpractical open loop circuit design. For example, just a single phantom measurement with an open loop configuration can easily produce tens of liters of radioactive wastewater (given a cardiac output of about 4 L/min), while the current setup reuses a volume of about 5 L for multiple measurements. Two sizes of silicone tubing (*Ø*_inner_ = 10 and 5 mm) are used to connect the individual components in the setup, distinguishing between higher and lower flow values.

## Methods

### Phantom measurements

In total, twenty-two phantom measurements were executed spread over six, non-consecutive measurement days. Table 1 provides an overview of the phantom and flow circuit variables in our measurement protocol. The measurements performed in this study were aimed for performance evaluation and optimization. As can be seen, the first fourteen measurements were used to optimize the overall setup and the remaining eight measurements were executed using the same phantom configuration. Results from the optimized setup (e.g., comprising an adequate myocardial tissue configuration) were used for further data analysis.

**Table 1:** Measurement variables

ID	Phantom variables						Flow circuit variables					
	*s*_MYO1_	*m*_s,MYO1_	*s*_MYO2_	*m*_s,MYO2_	*s*_MYO3_	*m*_s,MYO3_	*Φ*_AI_	*Φ*_MYO1_	*Φ*_MYO2_	*Φ*_MYO3_	*P*	Filters
		(g)		(g)		(g)	(L/min)	(mL/min)	(mL/min)	(mL/min)	(%)	y/n
D1#1	AC + Z	30	AC + Z	15			4.5	100	100	100	6.7	n
D1#2	AC + Z	30	AC + Z	15			4.5	100	100	100	6.7	n
D2#3	AC + Z	30	AC + Z	15			4.0	100	100	100	7.5	y
D2#4	AC + Z	30	AC + Z	15			4.0	100	100	100	7.5	y
D2#5	AC + Z	30	AC + Z	15			4.0	50	100	100	6.3	y
D2#6	AC + Z	30	AC + Z	15			4.0	50	125	125	7.5	y
D3#7	Z	7	Z	7	Z	7	4.0	80	80	80	6.0	y
D3#8	Z	7	Z	7	Z	7	4.0	80	40	80	5.0	y
D3#9	Z	7	Z	7	Z	7	4.0	80	0	80	4.0	y
D3#10	Z	7	Z	7	Z	7	4.0	80	40	80	5.0	y
D4#11	AC + Z	20	AC + Z	20	AC + Z	20	4.0	80	80	80	6.0	y
D4#12	AC + Z	20	AC + Z	20	AC + Z	20	4.0	80	80	80	6.0	y
D4#13	AC + Z	20	AC + Z	20	AC + Z	20	4.0	40	40	40	3.0	y
D4#14	AC + Z	20	AC + Z	20	AC + Z	20	4.0	80	0	80	4.0	y
D5#15	AC	7	AC	7	AC	7	4.0	80	40	80	5.0	y
D5#16	AC	7	AC	7	AC	7	4.0	40	80	40	4.0	y
D5#17	AC	7	AC	7	AC	7	4.0	40	40	40	3.0	y
D5#18	AC	7	AC	7	AC	7	4.0	80	80	80	6.0	y
D6#19	AC	7	AC	7	AC	7	4.0	40	40	40	3.0	y
D6#20	AC	7	AC	7	AC	7	4.0	80	80	80	6.0	y
D6#21	AC	7	AC	7	AC	7	4.0	80	80	80	6.0	y
D6#22	AC	7	AC	7	AC	7	4.0	40	80	80	5.0	y

*D* day, *s* sorbent, *m* mass, *AC* activated carbon, *Z* zeolite, *MYO* myocardial segment, *AI* arterial input, *Φ* flow, *P* perfusion rate

Performance was evaluated regarding to: (1) software compatibility, and (2) mimicking of radiotracer kinetics. Software compatibility was investigated by loading all phantom image data into the clinical software. We have verified whether the software would consider the phantom as a human heart and whether all processing and analysis steps can be performed sufficiently. Mimicking of radiotracer uptake/retention was explored empirically. We have filled the myocardial segments with two types of sorbents, namely activated carbon, and zeolite (SuperFish, biological filter media, Aquadistri, Klundert, The Netherlands), in different compositions (see Table 1 phantom variables). Regional and global perfusion deficit was mimicked by halving the standard volume flux of 80 mL/min, representing normal perfusion, in one or all three myocardial segments (see Table 1 flow circuit variables).

### Myocardial perfusion imaging

**Fig. 3:**
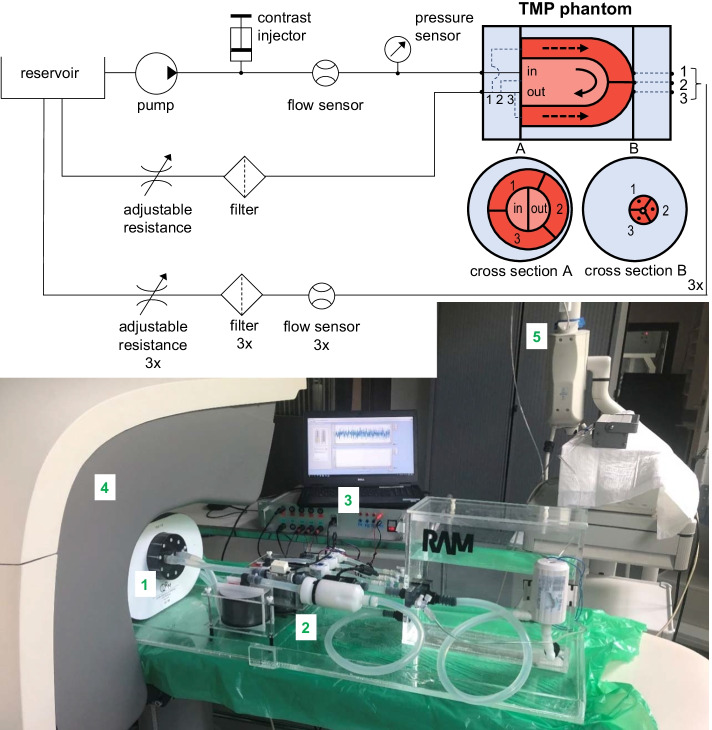
Fluid circuit diagram of the Twente Myocardial Perfusion (TMP) phantom setup (top) and subsequent experimental setup during performance testing (bottom). The numbers represent the following components: 1. the myocardial perfusion (and thorax) phantom, 2. its accompanying fluid circuit, 3. in-house built hard- and software for pump steering and sensor readout, 4. dynamic SPECT scanner and 5. the contrast injector

Phantom measurements included dynamic imaging of the flow of a radiotracer bolus through the LVC and myocardial segments over time. Figure 3b presents an overview of the experimental setup during performance testing with a clinical cadmium-zinc-telluride SPECT system (D-SPECT, Spectrum Dynamics, Caesarea, Israel). The TMP and thorax phantom were positioned in the scanner’s field of view in a standard way. All flow circuit variables were set prior to dynamic MPI acquisition and were kept the same for the entire scanning period. The clinical dynamic MPI acquisition protocol consisted of two 6 min dynamic scans, namely a rest scan followed by a stress scan, respectively. The phantom cannot distinguish between these patient-related physiological states, though the additional scan can be used for another purpose. During the rest scan, no radiotracer was administered. This baseline scan was solely used for background subtraction of previously trapped radiotracer. The subsequent dynamic stress scan started just before injection of the radiotracer bolus. 1.5 mL of 500 MBq ^99m^Tc-pertechnetate solution was injected by a clinical contrast injector (Mark V Provis, Medrad, Warrendale, USA) at 1.0 mL/s, followed by a 40-mL saline flush.

After scanning, the orientation of the imaged heart contours was manually adjusted using vendor software. We applied the same rotation angles [i.e., 0° along the sagittal axis (SA) and 90° along the vertical long axis (VLA)] for all scans since the phantom was positioned under the scanner in a standard way. The list-mode image data was re-binned into 32 frames consisting of 21 frames of 3 s, 4 frames of 9, 15, 21 and 27 s, and 7 frames of 30 s. An ordered subset expectation maximization technique was used for reconstruction of the dynamic image acquisitions with 4 iterations and 32 subsets [19]. Detailed information on the clinical workflow with a D-SPECT scanner can be found in published studies [20, 21].

### Image processing

Resulting dynamic image datasets were processed and analyzed with clinical software (Corridor4DM software, INVIA Medical Imaging Solutions, USA). These images contained a total number of pixels of 9216 (96 × 96) with a pixel size of 2.26 mm × 2.26 mm. In this software, myocardial areas were estimated from summed myocardial image cross sections [19]. We indicated preferred dimensions of the myocardial areas in a way they would match in length and width with all phantom measurements. The software included these dimensions subsequently in its calculation of the myocardial contours. The contoured time-lapse image data was displayed as cross sections along the short axis (SA) (from base to apex), horizontal longitudinal axis (HLA), and vertical longitudinal axis (VLA). Summed myocardial images were also captured in a polar map.

**Fig. 4:**
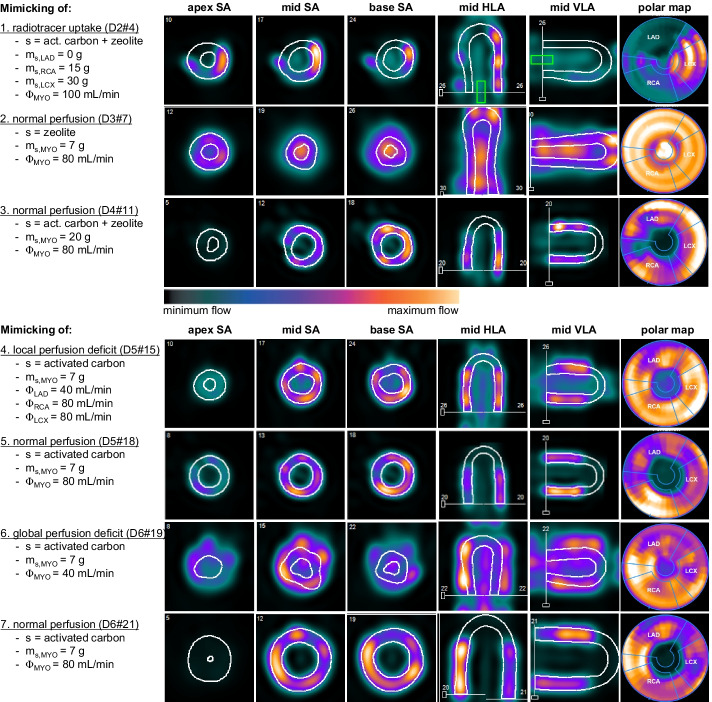
A selection of seven myocardial perfusion imaging series aiming to highlight the overall phantom development and evaluation process. The image cross sections and polar maps present the accumulated radiotracer distribution inside the phantom, which is a static representation of a dynamic measurement. Note that the relative color scale is determined by default for each individual measurement and is therefore not linked between measurements. In these measurements, mimicking of radiotracer uptake, normal perfusion and perfusion deficit was examined using different parameter settings. Settings include the type and mass of sorbent (*s*) utilized to mimic myocardial tissue, and the amount of volume flux (*Φ*) directed through the myocardial segments (MYO). D2#4 stands for the measurement day and number, respectively. *LAD* left anterior descending coronary artery, *RCA* right coronary artery, *LCX* left circumflex coronary artery, *SA* short axis, *HLA* horizontal long axis, *VLA* vertical long axis

Based on these contoured images, regions of interest (ROIs) were drawn automatically at the center of the LV base (see default size green box in Fig. 4 example 1) and within the myocardial surfaces. We used the American Heart Association (AHA) 17-segment heart model for standardized segmentation of the myocardial surfaces [22]. In this heart model, segments 1, 2, 7 and 8 corresponded to the mimicked LAD region, segments 3, 4, 9 and 10 to the RCA region and 5, 6, 11 and 12 to the LCX region. When the image data was correctly aligned, these displays matched with the three myocardial segments within the TMP phantom. Segments 14–17 were excluded in this analysis as the phantom does not mimic the apex.

In the vendor software, the average measured activity over time within these ROIs was displayed in time activity curves (TAC). Two arterial input functions (AIFs) and multiple tissue response functions (TRFs) were acquired per phantom measurement since each measurement comprised of two dynamic scans (rest and stress). Background activity, as present in the TACs derived from the rest scans, was then subtracted from the stress scans using a standard tool in the software. The resultant TACs served as input for myocardial blood flow estimation.

### Myocardial blood flow estimation

Myocardial perfusion or myocardial blood flow (MBF) is generally expressed as the flow rate normalized by the mass of the tissue volume of interest (in mL/min/g). The blood flow model applied in the clinical MBF analysis software was a net retention model proposed by Leppo et al. [23] and Yoshida et al. [24]. The following modified equation was used to calculate the retention rate (*R*) of tracer in the myocardium in mL/g/min. This myocardial uptake was expressed as the product of the MBF (mL/g/min) and the extraction fraction (*E*):

$$R = {\text{MBF}} \times E = \frac{{\frac{1}{{{\text{PV}}\left( {t_{3} - t_{2} } \right)}}\int_{{t_{2} }}^{{t_{3} }} {\left( {P\left( t \right) - S_{{\text{m}}} C_{{\text{a}}} \left( t \right)} \right){\text{d}}t} }}{{{\text{CF}}\int_{0}^{{t_{1} }} {\left( {C_{{\text{a}}} \left( t \right) - S_{{\text{b}}} P\left( t \right)} \right){\text{d}}t} }}$$

In this, *C*_a_(*t*) and *P*(*t*) corresponded to the average arterial and tissue tracer concentration over time, the AIF and TRF, respectively. Integration limit t_1_ denoted the end of the blood pool phase and was set by default to 60 s. *t*_2_ and *t*_3_ denoted integration limits of the average tissue activity, which was set to 60 and 120 s, respectively. Several predefined corrections by the software were applied to the data to compute absolute flow. Firstly, the acquired myocardial counts were corrected for partial volume losses using a recovery coefficient (PV) for the myocardium (PV = 0.63). Partial volume effects regarding measurement in the blood pool activity were neglected, therefore the cross-calibration factor (CF) was 1. Finally, theoretically computed spill over fractions *S*_m_ and *S*_b_ were set at 0.4 and 0. These fractions corrected for spill over from the blood pool activity to the myocardium and vice versa [25–27].

### Data analysis

All visual MPI data was analyzed in the clinical software (see image processing). Derived TACs were exported to MATLAB (MathWorks, R2021b) for further analysis. In this, all TACs were first normalized based on their injected activity (ranged between 468 and 551 MBq, normalized to 500 MBq). Then, linear interpolation was performed prior to peak alignment of the AIFs. Peak locations were determined using a spline interpolation technique. The aligned timescale was then also applied to the TRF data. Subsequently, the mean and standard deviation (SD) of corresponding AIFs and TRFs were plotted in combination with the mean and SD of specified area under the curves (AUCs). The AUCs comprised of the integration limits as denoted by the used blood flow model. Myocardial blood flow estimation by the clinical software was compared to ground-truth volume flux measurement and visualized in a polar map. In this, the set volume flux of 80 mL/min went through a tissue volume of interest of 53 ± 2 mL. Assuming a tissue density of 1 g/mL, this resulted in a ground truth, normal myocardial blood flow of 1.5 mL/g/min.

## Results

A selection of seven myocardial perfusion image series is displayed in Fig. 4 to highlight the overall phantom development and evaluation process. In 16 out of 22 phantom measurements, a similar orientation and alignment of the phantom-based heart contours were obtained. Semi-automatic heart contour recognition failed in the experiments where sorption of radiotracer was lacking. The first example MPI series illustrate that the more sorbent utilized for myocardial segment fabrication, the more radiotracer trapping occurred. In the second example, only zeolite was used as sorbent, which led to less overall accumulated adsorption of radiotracer compared to a similar phantom measurement executed with activated carbon (see examples 4–7). After thirteen setup optimization measurements, 7 g of activated carbon was found to be sufficient for the manufacturing of each myocardial segment, which was then applied in further phantom testing and evaluation.

Mimicking of normal perfusion, regional and global perfusion deficit was illustrated in examples 4–7. The clinical software could not detect the heart contours of a simulated global perfusion deficit. A local perfusion deficit was evidently visible in the MPI series, especially if it was the first measurement of the day and no radiotracer activity had yet accumulated in the segments of a previous measurement.

**Fig. 5:**
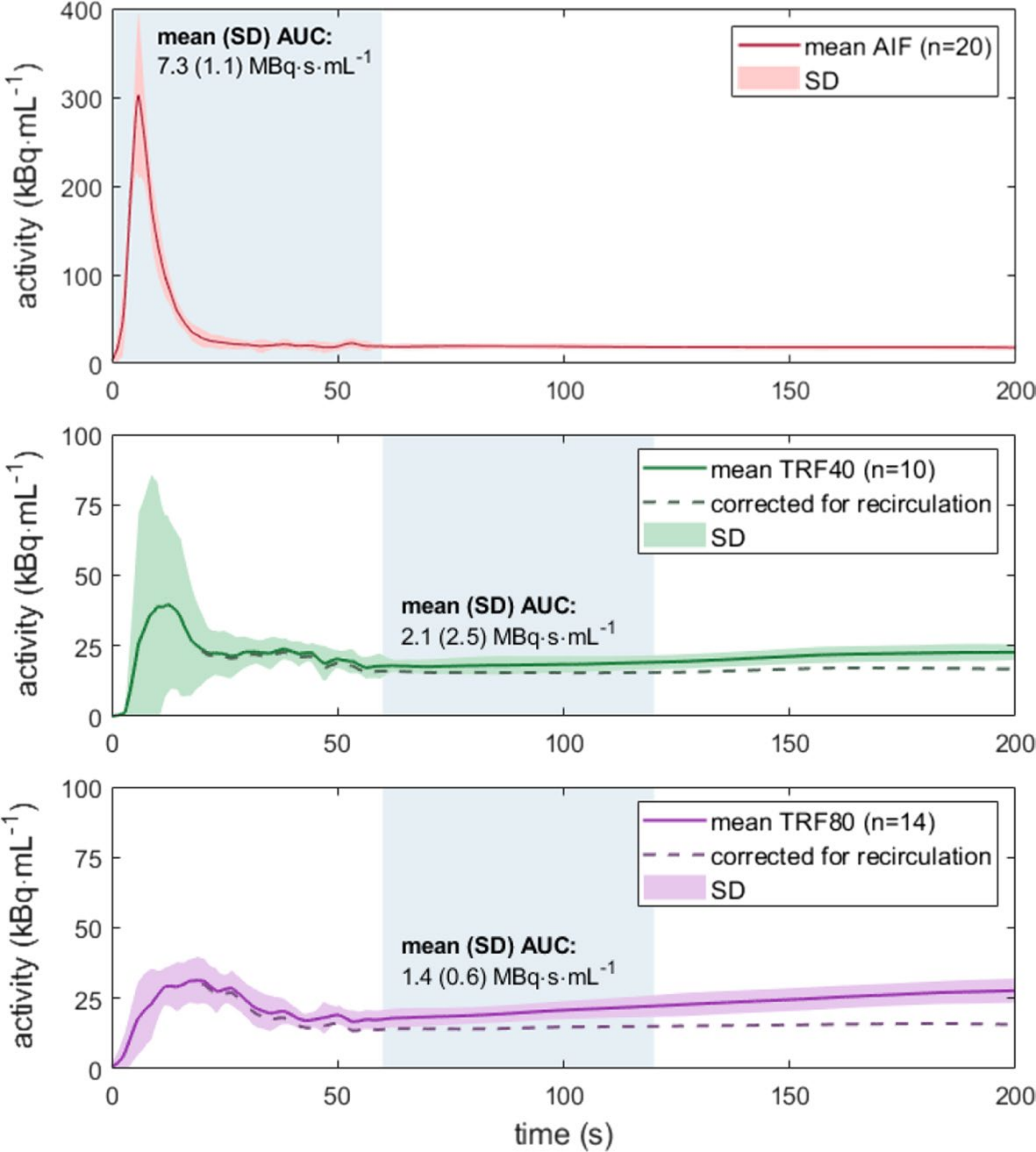
Time activity curves derived from phantom measurements. The mean arterial input function (AIF) and tissue functions (TRFs) of *n* measurements were plotted together with their standard deviation (SD). TRF analysis distinguished between myocardial segment flow settings of 40 and 80 mL/min. The AUCs within the indicated blue regions served as input for myocardial blood flow estimation. The dashed lines in the TRFs suggest how the curves would look when corrected for radiotracer recirculation

TACs derived from these dynamic MPI series are summarized in Fig. 5. The mean AIF and SD of 22 measurements was plotted, together with its mean AUC and SD. A similar analysis was shown for the TRFs that corresponded to a volume flux of 40 and 80 mL/min. In addition, a noteworthy, increasing tracer activity was visible in the TRFS over time.

**Fig. 6:**
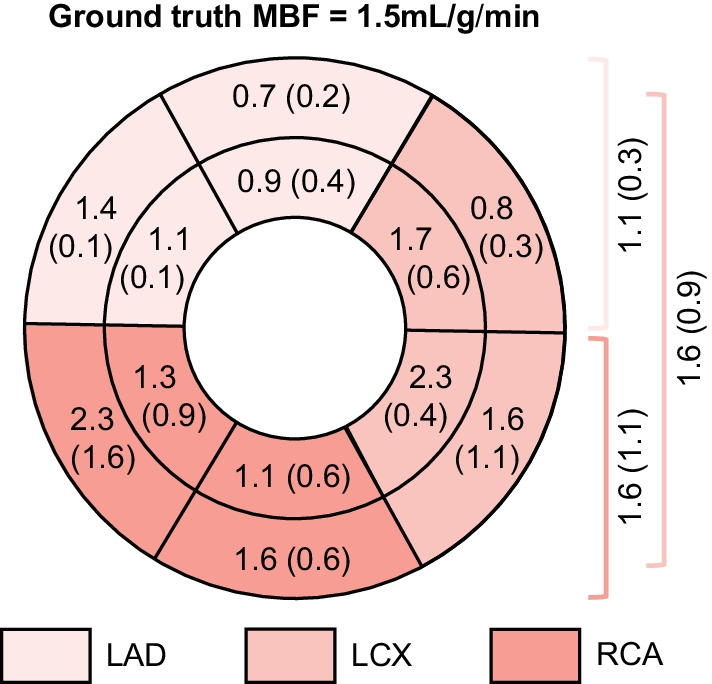
Preliminary results of computed MBF by clinical software (presented in polar map) compared to ground-truth phantom flow measurement. The presented mean values and standard deviations (SDs) are in mL/g/min. The inner segments of the plot are left blank as the phantom does not mimic the apex. On the right, the mean MBF estimates and SDs are listed for the mimicked regions supplied by the left anterior descending coronary artery (LAD), the left circumflex coronary artery (LCX) and right coronary artery (RCA)

An absolute value for segmental MBF was estimated for phantom measurements with a volume flux of 80 mL/min. These preliminary results are plotted in Fig. 6. As shown, the mean MBF for the twelve heart segments ranged between 0.7 and 2.3 mL/g/min. The absolute mean error between the computed MBF and ground truth was 0.4, 0.1 and 0.1 mL/g/min for the overall mimicked LAD, RCA and LCX region, respectively. Additional file 1: Video 1 illustrates the software display containing all analyses for measurement sixteen, including the time-lapse MPI data, TACs and regional MBF estimates.

## Discussion

This experimental work reflects on the design and realization of the TMP phantom, and on initial performance testing in dynamic SPECT-MPI. Performance testing was used to examine whether the set design goals were achieved. The first goal was to develop a phantom that is applicable/compatible with clinical MPI software. The second goal was that within the phantom, tracer kinetics can be mimicked up to the level of ground-truth comparison with software-derived MBF estimation. Results attained in both areas are now further described.

### Software compatibility

Compatibility was verified by going through all processing and analysis steps in the clinical software using the phantom data. It was possible to load all dynamic phantom MPI data into the 4DM program. In sixteen out of 22 measurements (73%), the myocardial contours were drawn correctly, which also implied that the delineated coronary regions and twelve heart model segments matched with the phantom orientation. Semi-automatic heart contour recognition failed in the measurements where sorption of radiotracer was lacking, hence mistaking accumulated tracer activity in the LVC for myocardial tissue (see example 1, 2 and 6 in Fig. 4). Contour delineation sometimes failed (partially) in cases where local and global perfusion deficits were mimicked. In general, inadequate delineation of poorly perfused tissue is also observed in clinical practice. However, the chosen 40 mL/min indicates a severe perfusion deficit. A global perfusion limitation of this magnitude is not patient realistic and was therefore not adequately analyzed by the software.

Hereafter, TAC analysis took place. A background subtraction is necessary to correct for already present radiotracer activity from previous measurement(s). A 1-day clinical protocol for dynamic rest and stress MPI also requires this software feature for the same reason [28]. Unfortunately, the usefulness of this clinical feature could not be further examined because of a small remaining percentage of recirculating tracer over time. The mean TRFs in Fig. 5 highlight this effect, whereby a suggestion has been made on the expected course in the absence of tracer recirculation (assuming a linear increase starting from ~ 20 s). This is also a partial explanation for the relatively high SD observed in similar/reproducible TACs. The highest SDs were observed in the first 60 s of the mean TRF40 (Fig. 4). This variation is the result of misalignment of the heart contours, hence mistaking accumulated tracer activity from the AIF for myocardial tracer activity.

Despite of the recirculation drawback, we continued our analysis and showed how ground-truth comparison between measured volume flux of normal perfusion (flow sensor readout) and software-derived MBF estimation could be envisioned using the phantom setup (Fig. 6). Yet, before such data can be interpreted, the measurement setup must be further optimized first.

Overall, compatibility with other commercial MPI analysis software is expected as well, as the phantom resembles a human heart sufficiently. However, the results in MBF quantification may differ [29]. This is one of the reasons why it is important to include various software packages in future research, for example, to define similarities, differences and boundary conditions (e.g., by exposing the software to phantom measurements covering the full range of myocardial perfusion levels).

### Mimicking tracer kinetics

Now we have shown that the clinical software can be successfully applied for evaluation purposes of the phantom setup, the next step is to evaluate the extent to which the TMP phantom can mimic the desired tracer kinetics. This study makes use of sorbents, whose mode of action is very similar to the physiological processes of (temporary) tracer retention in the myocardial tissue. The first pass extraction fraction of ^99m^TC-labeled MPI tracers is around 55–65% of the total injected dose, and for a small fraction (1–5%) irreversibly trapped in myocardial tissue [30]. As shown in example 1 in Fig. 5, the degree of simulated radiotracer trapping depends on the type and amount of sorbent used. Activated carbon seems to be a suitable sorbent for this tracer application, as it also shows irreversible trapping. When using zeolite, we have observed reversible trapping, which may indicate that the zeolite acts as an ion exchanger instead of an adsorbent. Remarkably, there is no homogeneous distribution of accumulated tracer within the myocardial segments. This may be because the sorbents used are granulates with dimensions up to ~ 5 mm diameter. The empirically determined sorbent composition for adequate myocardial uptake simulation (7 g of activated carbon) covered only a small part of the total segment filling and was supplemented with plastic beads. We aim to achieve a better distribution of sorbent within the myocardial segments in future phantom research.

**Fig. 7:**
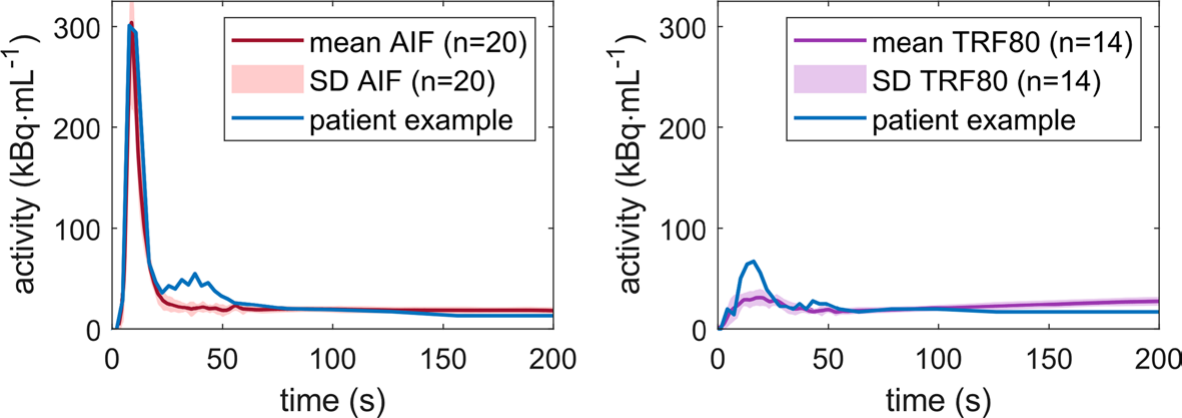
Time activity curve comparison of average phantom data (± SD) with patient example (derived from [33]). *AIF* arterial input function, *TRF80* tissue response function at a volume flux of 80 mL/min

To our knowledge, this tracer specific way of tissue mimicking has not been described before in the literature. Most perfusion phantoms (commercial and research oriented) use aligned porous fibers or 3D printed capillaries instead [11, 14, 31, 32]. In terms of transport characteristics, these fiber phantoms are solely based on convection and diffusion processes. Porous, capillary media can slow down processes like tracer retention but cannot realize actual trapping. The TMP phantom has the potential to exert more influence on mimicking perfusion characteristics as described in single- and multi-tissue compartment models. In line with this, we have observed satisfying similarities when comparing our phantom data with patient data (see Fig. 7). The visible differences come from spillover effects in the patient data (in the AIF at *t *≈ 30 s and in the TRF at *t *≈ 15 s) and the previously discussed tracer recirculation in the phantom TRFs. The latter results in a slightly rising phantom TRF over time, instead of a flattened line as observed in the patient example. The observed stronger increase in measured tracer activity in the patient TRF, including a higher peak activity, might also occur due to a higher flow. Extensive comparison with patient data was beyond the scope of this study but is an important component for future validation of the TMP phantom.

### Study limitations

The main limitation of this study was that the recirculation filters did not yet result in total extraction of tracer from the fluid circuit before recirculation. In first tests, the filters worked properly, thus we no longer changed this for follow-up measurements. Afterward, it turned out to be somewhat insufficient, which had consequences on the reliability and reproducibility of the results. A new filter design will be incorporated in future phantom measurements. A second limitation of this study is that we used ^99m^Tc-pertechnetate (easy accessibility), while in patient MPI studies radioactive technetium is labeled with tetrofosmin or sestamibi. A different molecule implies different sorption characteristics. Prior to these TMP phantom measurements, we studied the effect-size of this in adsorption column experiments. The differences seem small, though for future phantom validation purposes we intend to utilize the clinically used radiotracers. Of note, perfusion deficits simulating ischemia or infarction were not implemented in the current phantom setup. This issue needs to be addressed in a next phantom iteration. Finally, it could be mentioned why we started phantom evaluation using SPECT, while PET-MPI is considered the clinical standard. This was due to easy accessibility.

### Toward phantom application

Now that the possibilities of the TMP phantom in simulating tracer kinetics have been explored, and initial phantom measurements have been successfully executed in dynamic SPECT-MPI, a logical next step is to optimize and validate the setup for radiotracer specific MBF estimation. In addition, the phantom setup can be extended and tested for multimodal, absolute MPI applications. In phantom validation, it is important to verify whether choices and simplifications made in the phantom design are justified for the intended application. For example, the decision was made to first realize a stationary flow phantom. It can be argued that current phantom design is overly simplified due to missing cardiac contraction and respiratory motion dynamics. However, an outstanding measurement reproducibility is preferred over the degree of realism, especially since it concerns early-stage development of a *validation* phantom. This simplification could have presented misleading results in terms of an underestimated quantification accuracy. A possible solution is to include a motion inaccuracy factor (to be determined from literature) during future phantom application. Moreover, even without these motion dynamics incorporated, the phantom has relevant application domains, e.g., in studying the effect of patient size on MBF computation independently from motion influences. Another simplification concerns that we excluded mimicking of the left ventricular apex. Phantom design incorporates three identical myocardial regions to have similar flow dynamics and tracer kinetics present in all three regions. This appearance mismatches clinical delineation of coronary regions and heart segments, as the apex falls entirely within the LAD region. We performed only TAC and MBF analysis in the more basal heart segments, and disregarded apex simulation, to prevent misleading data analysis. In next iteration phantom design, the apex will be incorporated. In future research, we do strive for phantom redesign in which the apex is incorporated.

As a final remark, large SDs were observed for the mean TACs, mean AUCs and mean MBFs. On the one hand, we expect to be able to maximize measurement accuracy and precision by further optimizing the phantom setup (based on the findings obtained in this study). On the other hand, it may also be the case that part of these obtained deviations fall within the uncertainty of the measurement technique used. Whether absolute SPECT-MPI is accurate and precise enough is a prime example of what we can study further in such controlled phantom environment.

## Conclusion

The presented myocardial perfusion phantom is a first step toward ground-truth validation of and harmonization between multimodal, absolute MPI applications in the clinical setting. The set phantom design goals have been largely achieved. Due to its dedicated and 3D printed design, we have facilitated tracer kinetic phantom measurements, including TAC and potentially compartmental MBF analysis using commercially available software.

## Supplementary Information

**Additional file 1**. Clinical software display of dynamic and quantitative myocardial perfusion image analysis.

## Data Availability

The datasets used and/or analyzed during the current study are available from the corresponding author on reasonable request.

## Abbreviations

AC: Activated carbon
AHA: American Heart Association
AIF: Arterial input function
AUC: Area under the curve
C_a_(t): Average arterial radiotracer concentration over time (in kBq/mL)
CF: Calibration factor
E: Extraction fraction
HLA: Horizontal longitudinal axis
LAD: Left anterior descending coronary artery
LCX: Left circumflex coronary artery
LVC: Left ventricular cavity
MBF: Myocardial blood flow (in mL/g/min)
MPI: Myocardial perfusion imaging
m_s_: Sorbent mass (in g)
myo_1-3_: Myocardial phantom segments 1–3
OSEM: Ordered subset expectation maximization
PET: Positron emission tomography
PV: Partial volume effect
*P*(*t*): Average tissue radiotracer concentration over time (in kBq/mL)
*R*: Retention rate
*s*: Sorbent
SA: Sagittal axis
SD: Standard deviation
*s*_b_, *s*_m_: Spill over fraction from blood pool or myocardial tissue
SPECT: Single photon emission computed tomography
*t*: Time
TAC: Time activity curve
^99m^Tc: Radioactive isotope of technetium
TMP: Twente Myocardial Perfusion
TRF: Tissue response function
*Φ*: Volume flux (in L/min or mL/min)
*Z*: Zeolite
